# Understanding recruitment and retention in the NHS community pharmacy stop smoking service: perceptions of smoking cessation advisers

**DOI:** 10.1136/bmjopen-2015-010921

**Published:** 2016-07-07

**Authors:** Ratna Sohanpal, Carol Rivas, Liz Steed, Virginia MacNeill, Valerie Kuan, Elizabeth Edwards, Chris Griffiths, Sandra Eldridge, Stephanie Taylor, Robert Walton

**Affiliations:** 1 Asthma UK Centre for Applied Research, Centre for Primary Care and Public Health, Barts and The London School of Medicine and Dentistry, Queen Mary University of London, London, UK; 2 Faculty of Health Sciences, University of Southampton, Southampton, UK; 3 University of Oxford, Oxford, UK

**Keywords:** Smoking cessation, Community Pharmacies, QUALITATIVE RESEARCH, Patient recruitment, Health Behaviour, Communication

## Abstract

**Objectives:**

To understand views of pharmacy advisers about smoker recruitment and retention in the National Health Service community pharmacy stop smoking programme.

**Design:**

Thematic framework analysis of semistructured, in-depth interviews applying the Theoretical Domains Framework and COM-B behaviour change model. We aimed to identify aspects of adviser behaviour that might be modified to increase numbers joining and completing the programme.

**Participants:**

25 stop smoking advisers (13 pharmacists and 12 support staff).

**Setting:**

29 community pharmacies in 3 inner east London boroughs.

**Results:**

Advisers had preconceived ideas about smokers likely to join or drop out and made judgements about smokers' readiness to quit. Actively recruiting smokers was accorded low priority due in part to perceived insufficient remuneration to the pharmacy and anticipated challenging interactions with smokers. Suggestions to improve smoker recruitment and retention included developing a more holistic and supportive approach using patient-centred communication. Training counter assistants were seen to be important as was flexibility to extend the programme duration to fit better with smokers’ needs.

**Conclusions:**

Cessation advisers feel they lack the interpersonal skills necessary to engage well with smokers and help them to quit. Addressing advisers' behaviours about active engagement and follow-up of clients, together with regular skills training including staff not formally trained as cessation advisers, could potentially boost numbers recruited and retained in the stop smoking programme. Adjustments to the pharmacy remuneration structure to incentivise recruitment and to allow personalisation of the programme for individual smokers should also be considered.

**Strengths and limitations of this study**

- This is the first study to provide insight into smoker recruitment and retention in the National Health Service community pharmacy stop smoking programme from the adviser's perspective and therefore fills an important gap in knowledge.
- We used the Theoretical Domains Framework and COM-B behaviour change model as a framework to identify adviser attitudes and behaviours that could potentially be modified, thus providing a unique insight into smoker recruitment and retention in this clinical setting and facilitating development of interventions.
- In our analysis, we achieved theoretical saturation, high inter-rater reliability and high agreement with a health psychologist about mapping thematic data onto constructs of the Theoretical Domains Framework.
- While recruitment and retention are clearly two separate behaviours, we analysed them together because we were interested in the combined result—namely increased throughput in the service. Future studies might profitably separate these behaviours to examine their unique motivations.
- Our findings will not be generally applicable, but could nevertheless be transferable to community pharmacies delivering the stop smoking programme in other socioeconomically deprived communities across the UK. Certain findings such as low self-efficacy in consultation skills and the need to train all pharmacy staff to increase service throughput may well be transferrable to other healthcare systems; however, this would need to be examined in the different settings.

## Introduction

Improving public health through extending community pharmacy services has become a key part of UK National Health Service (NHS) operating strategy.^1^ ^2^ Smoking cessation is a public health priority^3^ since smoking is the most important cause of premature morbidity and mortality worldwide.^4^ In the UK, smoking cessation through pharmacy-led stop smoking services is now a key element of the Department of Health's tobacco control strategy.^2^

Community pharmacies are accessed by people in health and illness; hence, community pharmacy stop smoking advisers are well placed to reach large numbers of smokers.^5^ Behavioural counselling/support or a combination of nicotine replacement therapy and counselling by trained pharmacy staff are effective in helping smokers to quit.^2^ ^4^ ^6^ However, the overall effectiveness of the pharmacy stop smoking programme does not only depend on the quit rates achieved and also on the number of smokers who participate and adhere to the programme.^7^ ^8^ In 2012–2013 of the estimated 2 million smokers in England,^9^ only 149 034 smokers set a quit date in the pharmacy NHS stop smoking programme. Of these, 48% successfully quit at 4 weeks compared to the target of 70%.^2^ ^10^ Quit rate was slightly lower than that achieved by the stop smoking services in primary care (50%) and lower than specialist stop smoking services (56%).^10^ This apparent poor performance may result from differences in case mix; however, variations in staff training and environmental factors in pharmacies may make high cessation rates harder to achieve than in other settings. Nevertheless, there are considerable disparities in quit rates between pharmacies suggesting potential for improvement.^2^

A recent review^6^ suggested that pharmacists only target those they perceive to be ready to quit, thus reducing recruitment potential. Retention within the service is also poor, for example, only 35% of the 4500 people who joined the stop smoking programme in one inner London borough successfully quit (ie, quit status biochemically verified by carbon monoxide testing at 4 weeks from the quit date)^11^ and the remainder were lost to follow-up.^12^ Thus, in addition to optimising quit rates, improving recruitment and retention to the stop smoking programme might raise overall numbers successfully giving up smoking.

In a systematic review of pharmacist views on delivery of public health services, pharmacists recognised that they should be more active in smoking cessation; however, a number of barriers were suggested such as lack of time, staff and a designated space. Knowledge and skills were thought to be lacking, and patients did not expect to receive health promotion advice from pharmacists. In addition, there was reluctance to initiate conversations about health promotion because of fears of generating negative responses.^13^

No detailed investigation of stop smoking advisers' views on factors affecting recruitment and retention in the NHS stop smoking service has been published to date. This is the first such UK qualitative study aiming to (1) understand stop smoking advisers' views relating to smoker recruitment and retention in the NHS stop smoking programme in community pharmacies and (2) identify factors that might be targeted in an intervention to increase smoker recruitment and retention to maximise the effectiveness of the programme.

The results of this study will contribute to development of a complex intervention to promote uptake and increase the effectiveness of the NHS pharmacy smoking cessation service. This intervention will be evaluated in a cluster randomised trial in east London as part of the STOP programme (Smoking Treatment Optimisation in Pharmacies).

## Methods

### Study design

We conducted semistructured, in-depth interviews with stop smoking advisers (comprising pharmacists and pharmacy support staff). For roles and responsibilities of staff and an outline of the stop smoking programme, see box 1.

Box 1 **Outline of the stop smoking training and the community pharmacy stop smoking programme**

*Training in smoking cessation*^19–22^

▸ The National Centre for Smoking Cessation and Training in National Health Service (NHS) England offers a range of training, assessment and certification programmes for clinical and non-clinical health and social care workers to become more skilled in smoking cessation. The training programme is built around evidence-based behaviour change techniques that provide an understanding of the factors involved in smoking and smoking cessation.

The training includes:

1. Level 1 training or Very Brief Advice in Smoking Cessation training—this (online) training enables promotion of smoking cessation and can be undertaken as a minimum by healthcare professionals, for example, doctors, nurses and pharmacists including non-healthcare professional staff who advise people on how to quit smoking.
2. Level 2 training or Training and Assessment programme—this (online and face to face) training is for equipping healthcare professionals, who intend to become stop smoking advisers, with knowledge and skills to provide intensive one-to-one support in smoking cessation through delivery of the NHS Stop Smoking Programme (see below).

*Delivery of the NHS stop smoking programme in community pharmacy*^23^ ^24^

▸ The pharmacy owners (contractors) are contracted by NHS England clinical commissioning groups and local authorities to deliver public health services including the NHS stop smoking services to meet the needs of the local population. The stop smoking services cover the range of activities from the proactive promotion of smoking cessation through to provision of the NHS stop smoking programme.

In community pharmacy, the stop smoking programme can be delivered by stop smoking advisers who might be:

- *Pharmacists*: qualified experts in the use of medicines for the treatment of disease. They offer a range of services such as Medicines Use Review and Prescription Intervention Service; New Medicine Service; Appliance Use Review Service; public health services, for example, stop smoking services, NHS Health Checks.
- Pharmacy support staff such as medicines counter assistant, dispenser, dispensing assistant, pharmacy technician and accredited checking technician who support the pharmacist in the selling of medicines and delivery of services.

*Content of the NHS stop smoking programme*^2^ ^20^

▸ The content includes delivery of behavioural support together with pharmaceutical treatments comprising nicotine replacement therapy (NRT), for example, patches, prescribed medication, for example, varenicline (Champix) or a combination of NRT and prescribed medication to help a smoker quit smoking.

▸ The duration of the programme, dependent on commissioners, ranges between 6 and 12 weeks with quit status to be recorded at 4 weeks, verified by carbon monoxide (CO) monitoring and sent to NHS England for reporting of statistics, monitoring and commissioning purposes.

Week 1

- Introduction and set planned quit date (∼½ hour meeting).
- Stop smoking adviser explains programme process.
- Gives service user/smoker information about three types of medication available (NRT, Champix (varenicline) tablets and Zyban (bupropion) tablets).
- Discuss which is most suitable for service user.
- Adviser takes and records service user carbon monoxide (CO) reading.

Weeks 2–4

- Brief meetings to check progress (each ∼¼ hour and informal). Quit status CO monitoring is recorded at week 4 for NHS England statistics.^2^

Week 5/6

- Longer meeting to discuss motivations and techniques to avoid relapse.

Weeks 6/7–12

- Programme available to service user, but the adviser is not obliged to follow-up.

We used thematic framework analysis^14^ ^15^ applying the Theoretical Domains Framework^16^ within the COM-B model of behaviour change.^17^ The Theoretical Domains Framework is synthesised from multiple behaviour change theories and comprises 14 domains of theoretical constructs that explain possible influences on behaviour.^16^ Each domain fits within one of the three components of the COM-B model—Capability Opportunity and Motivation.^17^ ^18^ For example, ‘Skills’ in the Theoretical Domains Framework maps on to ‘Capability’ in the COM-B model, ‘Professional Role and Identity’ to ‘Motivation’ and ‘Environmental Context and Resources’ to ‘Opportunity’.^18^ We used COM-B as a lens through which to view our data because it provides a practical basis for designing interventions aimed at behaviour change, helping to identify the behavioural target and the components of the behaviour system needing to be changed.^17^

### Setting and participants

The study was conducted in three inner east London boroughs: Tower Hamlets, Newham, and City and Hackney. These boroughs include south Asian and African/Caribbean communities with high levels of tobacco use and persistent health problems linked to social and economic inequalities.^12^ ^25^ ^26^ Smoking prevalence in these deprived boroughs is close to the UK national average (20%) or higher (21% in Newham, 23% in Hackney and 37% in Tower Hamlets).^12^ ^25^ ^26^ Purposive sampling was used to obtain a diverse range of views.^27^ We selected stop smoking advisers who differed by gender and duration of being an adviser. Independent community pharmacies were sent a letter and information sheet and contacted by telephone to arrange a face-to-face meeting in the pharmacy. Within pharmacies, the pharmacist usually suggested the adviser (usually themselves or a member of other pharmacy support staff, ie, stop smoking adviser for interview). A member of the research team (CR) or a research assistant obtained written informed consent.

### Data collection

Individual interviews were conducted from January to June 2014 by an experienced female qualitative researcher (RS and VMN) using an interview schedule (box 2). The interviewer was not known to the study participants and was not involved in recruiting participants to the qualitative study. Advisers were informed that study aim was to improve programme recruitment, retention and quit rates. Interviews took place in the consultation room of the community pharmacy and lasted 30–60 min. Recruitment and interviews continued until data saturation was achieved. All interviews were audiorecorded and fully transcribed. NVivo V.10 was used for organisation of data and to facilitate analysis.

Box 2 **Interview schedule**

*The consultations*

1. What helps/what do you do that makes smokers join the service (recruit)?
2. What helps/do you do that makes them keep coming back (retain)?

*Process*

1. Can you describe the main issues involved in delivering the stop smoking programme?
2. What prevents people from joining the stop smoking service?
3. What are the characteristics of people that do not join the service/who join the service and quit/who join the service and do not quit?
4. We know that many advisers select smokers most motivated to quit. What would make you take on the less motivated/interested? (This question was added after analysis had begun as an issue emerging from the data.).

*Training*

1. What would you like that is different to training you have had so far? (that can help more smokers to join the stop smoking service).

### Data analysis and interpretation

A thematic framework analysis^14^ ^15^ was conducted. This method starts deductively with a priori codes from the study aims and objectives however, subsequent analysis is inductive and grounded in the accounts of the participants.^14^ Researcher (RS) read and reread the transcripts for familiarisation and data immersion. A thematic framework was formed following line-by-line coding and comprised useful memos or descriptive statements to develop categories. Comparisons were made within and between transcripts based on the thematic framework. The data were then lifted and charted across the thematic categories. This process was carried out initially with a few transcripts and discussed with the study team (CR, RW and LS) to assess reliability and agreement between two coders and to check validity of the analysis. Inter-rater reliability was 90.9% (κ agreement) between the two coders (RS and CR) on 20% of the transcripts. Subsequently, the emergent themes were mapped (by RS) onto behavioural constructs of the Theoretical Domains Framework (TDF) and the COM-B model, where applicable, to generate analytical themes.

The mapping was discussed and agreed with a health psychologist (LS) to ensure the mapped thematic data fitted with the domain definition and its content (see online supplementary file 1). This process generated analytical themes or key domains influencing the engagement and the retention behaviour of advisers and helped us to identify domains that might be targeted to optimise adviser behaviour. Emergent themes are exemplified below with direct participant quotations.

10.1136/bmjopen-2015-010921.supp1 Supplementary data

## Results

Twenty-five interviews were conducted following approaches to 44 advisers in 29 community pharmacies. Reasons for non-participation were lack of interest (n=14) or unavailable/no answer (n=5). The participants were drawn from 15 pharmacies with 6 pharmacies contributing two or three interviewees. Over half of the advisers were of Asian ethnicity (56%) and a small proportion (20%) of pharmacies provided a multilingual service. Characteristics of the participants are shown in table 1. All participants were smoking cessation advisors trained to level 2.

**Table 1:** Characteristics of study participants

Study ID*	Pharmacist or support staff	Gender	Duration being stop smoking adviser
S11A101	Pharmacist	Female	Not given
S19A182	Pharmacist	Female	3 years
S08A071	Pharmacist	Male	Not given
S13A121	Pharmacist	Male	10 years
S29A281	Pharmacist	Male	4 years
S04A031	Pharmacist	Male	Not given
S09A081	Pharmacist	Male	10 years
S23A221	Pharmacist	Male	Not given
S25A242	Pharmacist	Male	7 years
S26A253	Pharmacist	Male	Not given
S03A021	Pharmacist	Male	Not given
S05A041	Pharmacist	Male	5–6 years
S11A103	Support staff	Female	9 years
S02A012	Support staff	Female	Not given
S25A241	Support staff	Female	1 year
S26A251	Support staff	Female	7 years
S09A082	Support staff	Female	5 years
S25A243	Support staff	Female	Not given
S26A252	Support staff	Male	4 years
S01A001	Support staff	Male	Not given
S04A032	Support staff	Male	6 years
S11A102	Support staff	Male	4 years
S19A181	Support staff	Male	12 years
S24A231	Support staff	Male	Not given

*This code is used to identify the source of quotations in the text.

**Figure 1:**
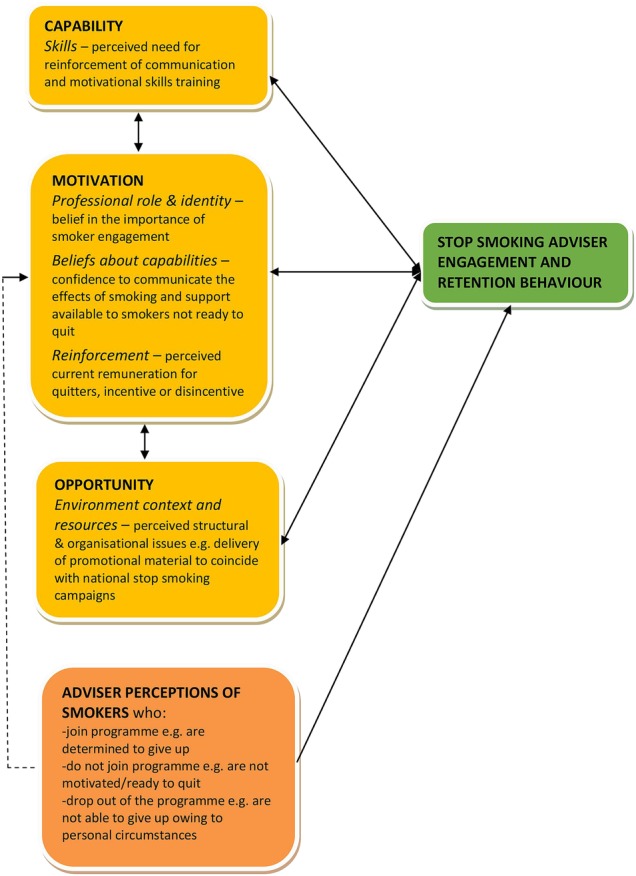
Illustration of behavioural factors affecting adviser engagement and retention behaviour (modified from the COM-B model).

In the analysis, the emergent themes mapped on to 5 of 14 TDF constructs with one independent theme. Figure 1 illustrates this using COM-B as headings and TDF as subheadings (italics). The independent theme and its potential relation to COM-B is highlighted with dashed lines. See supplementary file 1 for full definition of the COM-B and TDF constructs. In the text that follows, the higher level heading is from the COM-B model and the subheadings represent relevant domains from the Theoretical Domains Framework that explain two separate adviser behaviours, recruitment and retention.

### Motivation

#### Professional role and identity

Many participants felt proud of their work and were satisfied that through their role as advisers, they were able to help people quit smoking, which in turn helped with the health and well-being of the wider community.

I mean, you are always happy that you've helped someone do something. And there are many times that, this guy (client who has quit) will always pop in. …and say ‘Oh, I'm just passing and I thought let me just say hi to you!’ And there are many times … This lady, it's nice. I mean, why would this lady bring her son over for me to advise? It's like they appreciate it and you have that feeling that, oh, you've also managed to help someone and it's great to do that. (Study ID S08A071, Pharmacist)

##### Identification and engagement of smokers into the programme

Advisers identified potential clients through: general practitioner referrals; recommendations from people who had quit; opportunistically during medicine review or while conducting risk assessment; and when people bought products from the pharmacy, picked up regular prescriptions or bought over the counter medication. Despite this, many advisers selected and recruited only those smokers who mentioned their readiness to quit because they thought that these smokers were less likely to drop out. A few said they only recruited those who specifically asked for smoking cessation advice and were not interested in actively recruiting smokers to the NHS stop smoking programme (SSP).

She's like I'm not in the right frame of mind but I really want to give up smoking. So in that way we just tell them look, … So we told her and with smoking you have to make sure that they're in the right frame of mind otherwise there's no point them joining the programme. Otherwise they'll join and then quit after a week and there's no point. (S24A231, Pharmacy support staff)

I would say the recruitment. I mean it's a bit hard, most of it has to be walking and…yeah. Or maybe they see the poster. But we're not doing that much. (S19A182, Pharmacist)

Some participants said that recruitment into the programme was low priority. Advisers wanted to prioritise patients with long-term conditions or those that they thought had life-threatening conditions. One adviser felt giving up smoking was the responsibility of the smoker.

So smoking is more or less a very very little part of our actual work here. We actually have people who have life threatening conditions, you can be dead tomorrow. So that's of a greater priority than smoking. Smoking is by choice, you want to give up, you don't want to give up. (A01A001, Pharmacy support staff)

Several participants also said they believed that all the advice, support and products they provided could only help a smoker quit if the smoker was mentally prepared and had the willpower to follow through the advice and make the necessary lifestyle/environmental changes conducive to stopping smoking. Advisers felt they had most success with this type of client.

We're pretty straight with them (potential clients), … if they're motivated to quit then they've got a better chance of succeeding, things like …NRT Champed (sic) only helps to a certain extent … and if they don't have the willpower it's not going to work…. (S09A082, Pharmacist)

##### Encouragement and motivation to ensure attendance and adherence

Some participants stated that encouraging clients to continue attending the programme sessions was important. One adviser mentioned that to prevent dropouts and indeed when engaging with clients who had relapsed, identifying the reasons for stopping smoking and relapse were important.

Oh I think you are always going to get that (people dropping out). …I mean you are talking about 1 in 2 not getting through so you just have to keep motivating and telling them you know I mean you've been smoking for 20 or 30 years, you are not going to give up in 6 to 12 weeks, it's a long term process. And I think if you have like help, if you have re-started again, again the important thing is to find the reasons why because I think if you can get to the bottom of that and work on that rather than just giving them NRT and thinking, you know so really that is most important. The NRT helps but you need to know why you want to stop smoking. (S03A021, Pharmacist)

A few participants did not see following up clients who missed sessions as part of their role. Some said that they simply accepted it if smokers mentioned they were finding it difficult to not smoke when they spoke to them by telephone or in person.

Two persons I've seen, they can't manage (to quit), that's it. Sometimes if they come for their medicines to pick up or anything, they can say, ‘Oh, sorry, I didn't come back, because I can't manage at the moment, I'm not all right’. So we say, ‘OK, whenever you feel OK’. (S02A012, Pharmacy support staff)

When they don't come back it's like well, did you give them the wrong products? They didn't suit them, they could've phoned us, we could've spoken to them, we could've swapped it. We are pretty flexible. But if they don't come back to us there's no way of knowing, is there? (S25A241, Pharmacy support staff)

Some advisers acknowledged that more smoker engagement would result in higher quit rates. The following were suggested to boost service recruitment and retention: identify potential smokers for the service, for example, when they bought cough syrup; engage, inform, encourage and recommend the programme to smokers, for example, through use of poster adverts; explain how the programme and adviser involvement could help with quitting; and place a holistic focus on the person.

So if you're focused on the client rather than the process you're going to get a bigger outcome rather than saying this is a tick form, I've got to tick this, I've got to fill this in, I've got to fill that in and oh, I've missed out here, we've got to do a CO reading sort of thing. (S26A253, Pharmacist)

#### Reinforcement

##### Remuneration

This process of remuneration was acknowledged as an issue by the adviser participants who were a mix of owner pharmacists, employed pharmacists and other pharmacy support staff. Among the owner pharmacists, a few said that the current remuneration for quitters was reasonable, while some others felt that the remuneration did not take into account the time they spent with smokers encouraging them to join and adhere to the service.

One owner pharmacist mentioned that the current payment system was a disincentive to spend time with smokers, and needed revisiting by commissioners; payment based on quitters might influence which smokers staff are willing to support.

And things, we get penalised for non-quitters which are not really our fault.

Interviewer: …How do you mean penalised?

So currently we may spend three or four hours actually going through these patients, and because they don't quit we don't get the full £40 or £35. We end up with about £20. That's the disincentive. There are certain health authorities now are saying that if a patient doesn't quit there's no fees. So there is a lot of disincentives for a pharmacist to say look, I only get a 50 per cent success rate, I'm not going to spend three or four hours at a time and get nothing for it. So they're (pharmacies are) not going to take on new clients and the way that the health authorities and CCGs think about these programmes needs to change. (S26A253, Pharmacist)

Another owner pharmacist stated that perhaps smokers should be incentivised to attend their stop smoking appointments as it could mean saving money for the NHS in the long run and some pharmacy support staff suggested providing incentives/rewards to their clients that could be given at programme completion.

And I think if you can perhaps issue vouchers, but issue vouchers that are redeemable at the end of the process, yeah? So it makes no sense for them to come to two or three consultations, accumulate vouchers and then miss the fourth one, yeah, you know? So I think there should be vouchers that can be redeemed for cash at the end of it, because most people don't want to be restricted as to what they can do with a voucher or cash or anything. That is one thing I think…..at the end of the day, we need to put it in the balance; I mean, the government feels that getting people to stop smoking leads to a greater saving on the NHS, so it's something they should look at. (S29A281, Pharmacist)

Several of the employed pharmacists and support staff acknowledged that delivery of the programme was part of their job, although a few highlighted that if their pharmacy were to receive an increase in the current remuneration for quitters, then they might be allowed to spend more time engaging with and following up clients to continue and complete the programme.

I think so as well, because it (current remuneration) is a kind of barrier. You do encourage them (smoker) to stop smoking, but then three weeks you spend with them, although they don't stop smoking … And for one engagement they give you (payment), but after that, if they keep on giving us the money we (can) encourage more people, … So if they encourage me, I mean the pharmacy, by providing with more money or anything, no barriers regarding money, then we can do more, much more. (S26A251, Pharmacy support staff)

#### Beliefs about capabilities

##### Interaction with smokers

Some advisers mentioned that engaging with smokers and motivating clients who enjoyed smoking or who were not interested in quitting was a challenge to programme recruitment and retention. In addition, one participant was unsure about the protocol to follow when a client smoked a cigarette at the end of 12-week programme.

It's more of a challenge when they say oh, but I love smoking, I love the taste of smoking. Then it's a bit of a challenge because they've already got in their mind that…there's like a mental block. Whatever you're going to say to me, I don't really care sort of thing. .. (S24A231, Pharmacy support staff)

In contrast, a few participants felt confident to handle some of the above-mentioned issues by talking about the harmful effects of smoking, showing the tar jar to smokers and suggesting to smokers they were available to support them when they are ready to quit.

Yeah, I mean there's no harm in recommending it (the service). You recommend it to everyone. I don't see anything bad about that. It's just whether they'll actually sign up or not. … The only way we can deal with that is through awareness, like the tar outside and posters and our TV advert. That's the only way you're going to get those kind of people. (S26A252, Pharmacy support staff)

### Capability

#### Skills

##### Skills training

Several suggestions were given to improve the skill set of advisors (including counter assistants) to help with recruitment and retention in the stop smoking programme, such as reinforcement of motivational and communication skills, including how to approach people about smoking without causing offence and how to be supportive. Most suggestions were features of person-centred care which all healthcare professionals are expected to follow.^28^ These were: how to personalise advice, how to elicit from smokers which things had worked and which did not work, how to work with smokers to identify from them what targets they would like to achieve and how they would achieve them.

Pharmacies are very prescriptive and I think when you're asking people to give up smoking you have to think about how they need to change their behaviour, and I think it's those … skills that you need to apply, and that is very crucial and very important. Getting it out there. What is it they're able to do? You know what they need to do. It's only when they come up with solutions themselves they're more likely to follow it through. (S01A001, Pharmacy support staff)

In addition, a few participants stated that there was need for regular training, and some others suggested that it might be useful to include in any future training items such as visual scenarios, role plays or mock interviews to learn and practice their skills. Two participants also wanted to see experts in action with a client to help them learn and improve their skills.

I think sometimes you get a bit complacent. When you're doing it and you think everything's fine you give them the NRTs, tell them to come back next week if they have any problems. But they might actually have a bit of problems that they want to tell you but they can't for whatever reason or they feel like if they tell you that they've failed then… So trying to get the best out of their patients, so someone that could come in like a specialist to actually help, that would be good. Because you always learn something when you're shadowing someone, always. (S24A231, Pharmacy support staff)

### Opportunity

#### Environmental context and resources

The study participants mentioned a number of structural/organisational challenges that affected smoker recruitment and retention in the programme.

##### Lack of time because of delivery of other services

Participants mentioned that they forgot to remind clients about their appointment or they were unable to keep tabs on clients' programme adherence. A few pharmacists, on account of being busy, said they were reluctant to recruit smokers, if they were unable to provide details about the programme and the procedure involved; this seemed to affect recruitment.

Especially someone who says, “Can I join the scheme now?” and sometimes it's not possible; when I'm so busy here, I can't leave the place and come in here. There's no way I can leave it, so I have to give them an appointment. But sometimes people are not happy with that; it's like they really want to join now! You get the point, they really want to join, so if you can't do it for me now, then I can't come, so it's an issue. (S08A071, Pharmacist)

In contrast, one pharmacy support staff participant, to avoid losing clients, started engaging people on the same day of their pharmacy visit instead of giving them an appointment for a later date; this strategy helped them to improve recruitment into the stop smoking programme. Another staff member wanted a reminder system to enable client follow-up to help with retention.

If somebody comes in now, generally we do it straight away. That's how we found it works better. If you give them a date they don't turn up because within this time they've got a different commitment. (S11A102, Pharmacy support staff)

To make time for smoker engagement, one pharmacist mentioned that having counter assistants to help with the recruitment process or more staff to share with their dispensing workload could lead them to engage and offer the stop smoking programme more frequently to smokers.

##### Inflexibility of the programme

Several participants felt that the duration of the programme was insufficient for some clients to quit and remain abstinent. One participant suggested that clients who relapsed going through the programme should be allowed to continue with the programme instead of asking them to rejoin the programme, normally after a few weeks. In contrast, another participant mentioned that allowing dropouts to rejoin the programme as soon as they were ready instead of using the ‘after 6 months’ rule was not helpful in improving quit rates.

Other issues I've had have been people who kept coming back, like two or three times a year, and it's obvious that they are not serious, or I don't know. In the past, with the NRT, they used to say they can't do it again until after six months, but now as often as they want. But then with the Champix, I got people coming two or three times a year: stop/start, stop/start. (S11A103, Pharmacist)

##### Working with budget cuts

The facility to offer two treatments concurrently to clients had been stopped due to budget cuts despite evidence of its effectiveness. In one of the participating boroughs, the varenicline (Champix) license for pharmacists to prescribe had been taken away; one participant stated that this had affected recruitment into the programme.

Champix, the tablets. We haven't got a PGD (patient group direction) for it right now, so I had one person yesterday, she was a bit upset, she was like oh, I want the tablets, I want the tablets. And we don't have the licence for it. I think the whole of Newham, all the pharmacies…I think hopefully we'll be getting it back soon enough, … So that's a problem, we've lost a few people… but we could've had those people as clients. So that's one thing, hopefully we'll try and get that back. If the providers can help us with that then that would be great. (S24A231, Pharmacy support staff)

##### Failing to take advantage of national campaigns

One participant felt that the ‘Stoptober’ campaign helped with smoker recruitment but not retention because they perceived smokers were not really committed to giving up smoking and only joined because of the advertisement. Some other participants felt that the No Smoking Day campaign went by too quickly to enable smoker recruitment.

Having it (No Smoking Day) spread over a week means you can maybe put up a lot more balloons up, you can put a lot more signs up, you can market it better, it gives you a longer time to market it and it will give you that whole week to engage with people. Because sometimes you might only see them once a day, but if you've missed that opportunity…tomorrow's not a non-smoking day. Oh, I'm not doing it then. (S25A242, Pharmacist)

Other organisational factors such as inadequate stock of nicotine replacement products to meet client demand and having only one consultation room that was often occupied were mentioned as restrictions on smoker recruitment by two participants.

The room that I'm using is constantly being used up. I've got this one room. You know? And things like that again—having resources [unclear] how to manage that. (S01A001, Pharmacy support staff)

#### Advisers' perceived characteristics of service ‘joiners’, ‘non-joiners’ and ‘dropouts’

Advisers' characterised smokers as those likely to join, not join or drop out of the stop smoking programme. This categorisation of smokers then affected how likely the adviser would be to recruit them into the stop smoking programme.

Eleven participants stated that ‘Joiners’ (including those who relapsed and wanted to quit again) were those who were motivated, willing or determined to give up smoking to improve health/quality of life and were clear of their reasons for wanting to give up.

Yeah, always ask them as well what's in your mind. Why do you want to do this (quit smoking)?…So once they have that (reasons) in mind I will tell them okay, so have that in mind, so even when the cravings come just hang onto that, because at least you have something you want to achieve because of that. … in fact, if you don't have it, for me you are more likely to fail…. (S19A182, Pharmacist)

‘Non-joiners’ were characterised by eight participants as those who did not suffer from any health-related problems and hence were not ready or motivated to quit, they were either not interested or in denial, thinking that they would not suffer any health-related consequences perhaps because people they knew had been smoking for years and their health seemed unaffected. In contrast, smokers living with a long-term condition such as cancer felt that it was too late for them to change their behaviour as their illness progressed.

Because like I said, in many years, I've seen so many patient …, when they're smoking, end up with a cancer. He was still coming to see me, went to the doctors, diagnosed cancer, came in and said I'm not doing it (stopping smoking). Why? Because I've got cancer, what the hell, I'm going to die. (S19A181, Pharmacy support staff)

‘Dropouts,’ according to seven participants, were those who were fighting more than one addiction or going through some personal crisis and therefore were not ready to give up smoking. Such people, the participants suggested, might be easily tempted into smoking by family/friends who smoke, or might not perceive any benefits or might not be interested in quitting.

He was determined (to quit) because he got it from the GP, the letter, so I thought yeah, he's going to give up smoking. But he just…the first week I supply and then the second week I didn't see him there. Called him and he said no, I can't give up, it's not holding me now. So he wasn't ready really, I don't think he was ready. …. (S04A032, Pharmacy support staff)

## Discussion

### Statement of principal findings

Recruitment and retention of smokers in the NHS stop smoking programme may be influenced by advisers' preconceived ideas about smoker types likely to join, not join or drop out of the programme. This early categorisation of smokers influenced perceived readiness to quit. Active smoker recruitment was often a low priority, partly because advisers considered the remuneration that the pharmacy received for each quitter was insufficient to justify use of time and because they anticipated challenging interactions with some smokers. There were also perceived structural/organisational challenges involving programme delivery.

To improve the programme, advisers suggested that they should adopt a more holistic and supportive approach. They further suggested that strengthening their belief in the importance of engagement with new and relapsed clients would help to improve uptake of the programme and retention of smokers within the service. An increase in pharmacy remuneration for quitters was also thought to be beneficial. Advisers would welcome improvement in structural and organisational delivery of the programme and more regular training in person-centred communication including for counter assistants.

### Comparison to other studies

The Theoretical Domains Framework and COM-B models have been used previously in studies of dental health professionals^29^ ^30^ and midwives.^31^ A barrier to delivering smoking cessation services in these clinical settings was that healthcare workers did not consider smoking cessation part of their primary role. In contrast, all participants in our study were trained stop smoking advisers and wholeheartedly embraced the smoking cessation role. Nonetheless, some advisers identified the need for more training, particularly to build capability in the recruitment process, to maximise engagement with smokers and to bolster their communication skills. This need for training was also felt by dental health professionals and midwives. The fear of negative patient response, inadequate staffing and lack of confidence in delivery of health behaviour advice which we identified in our study has also been reported in other settings.^13^ ^32^ ^33^

In line with the recent review on the effectiveness of pharmacy smoking cessation interventions,^6^ we found that many advisers were mostly accepting smokers who wanted to quit because they did not want to risk dropouts. The National Centre for Smoking Cessation and Training also recommends that if the smoker is not ready to make a serious quitting attempt, the adviser should provide the programme's contact details and ask them to get in touch when they are ready.^34^ Another explanation for selection of those likely to quit may be pressure from service targets.^35^

The perceived opportunity cost of engaging with smokers who did not express a desire to quit or who fitted stereotypes that advisers associated with failure to quit appeared to affect adversely the recruitment of smokers into the service. Hoving *et al*^36^ compared ‘active’ recruitment (ie, ‘asking each individual's smoking status and inviting smokers to participate in the service’) and passive (ie, ‘leaving participation up to the smoker's initiative’) in a community pharmacy setting. The former approach was suggested as a way of increasing the total number of quitters. It has been suggested elsewhere that passive recruitment strategies might reduce the likelihood of a quit attempt.^37^

In addition to lack of time for health promotion which has been reported previously,^13^ ^32^ ^33^ ^38^ problems with delivery of medicines and promotional material, linked to national stop smoking campaigns, were perceived by advisers to affect smoker recruitment and retention. Addressing these issues is crucial to facilitate adviser engagement behaviour^39^ ^40^ and could add to the success of national campaigns in increasing the number of people attempting to quit and permanently quitting.^39^ ^41^ ^42^

### Implications for clinical practice and policymakers

Advisers' perceptions of types of smokers who are likely not to join or to drop out of the programme and some advisers' experience of challenging interactions with smokers have been reported previously^33^ including in socially deprived areas.^43^ ^44^ However, the community pharmacy stop smoking services in east London are effective in reaching socioeconomically deprived communities^35^ and the smokers in these communities are just as motivated to quit smoking as smokers in more affluent areas,^35^ ^45^ and to want help with quitting.^46^ Smokers' stage of change^47^ and economic disadvantage should not preclude the use of active recruitment^8^ ^35^ ^45^ and retention strategies.

Recent changes to the pharmacy smoking cessation services in one of the participating boroughs meant that pharmacists were no longer able to recommend cotherapy (nicotine patch plus gum/lozenge/inhalator) or varenicline to clients which advisers considered a problem. It seems likely that restricting access to effective treatment and thus compromising patient choice could adversely affect engagement of smokers and quit rates. In addition, several advisors felt the duration of the stop smoking programme was not long enough for some smokers to quit and to remain abstinent and suggested having flexibility in the programme to allow continuity of care, where appropriate to suit clients' needs, for example, after relapse.

The remuneration for programme delivery is determined by commissioners on the basis of clear criteria, for example, the time and duration of intervention and treatment provided, carbon monoxide monitoring and data reporting. The payment is made to the pharmacy owner, that is, a contractor who may be a pharmacist sole trader, a partner with other pharmacists or who may appoint a superintendent pharmacist^2^ ^48^ No payment is made to employed pharmacists or to pharmacy support staff. The current payment system for pharmacies appears to provide a disincentive to spend time with smokers—and this has been acknowledged in the community pharmacy stop smoking services guidance.^2^

The pharmacy cessation advisers derived professional satisfaction in helping individual smokers to quit and also saw their role in smoking cessation as benefitting the whole community. Advisers thought about wider aspects of the scheme and took a professional interest in how the scheme might develop—for example, suggesting a voucher scheme and tailoring the duration of the smoking cessation programme to individual smokers. This source of intrinsic motivation may distinguish cessation advisers from other healthcare professionals and could be drawn upon further in pharmacy adviser training programmes.

### Implications for future research

More research is needed with a broader range of participants including female advisers and advisers of other ethnicities delivering the programme in deprived and non-deprived areas. In addition, studies to understand in more detail the effects of the remuneration structure for pharmacists and support staff are warranted. We took recruitment and retention together in the analysis since we were interested in the joint outcome of these two behaviours, which leads to an increase in the total number of people completing the smoking cessation programme. While this seems justified given the purpose of our work, this is a potential limitation of our study since clearly the two behaviours may have different antecedents. Future work could usefully examine recruitment and retention separately leading to individual insights into these behaviours and how they might be modified to strengthen the NHS smoking cessation service.
