# Supplementary material for: Understanding recruitment and retention in the NHS community pharmacy stop smoking service: perceptions of smoking cessation advisers
Source: BMJ Open. 2016 Jul 7;6(7):e010921. doi: 10.1136/bmjopen-2015-010921 (PMC4947750; doi:10.1136/bmjopen-2015-010921)
Supplement: Supplementary data [file bmjopen-2015-010921supp.pdf]

Supplementary file 1 Theoretical Domains Framework (TDF) - Domain definitions

TDF (definition)	Theoretical Constructs	Possible Example
**Knowledge**<br>*An awareness of the existence of something*	Knowledge (including knowledge of condition /scientific rationale); procedural knowledge; knowledge of task environment	Knowledge of why important to ask people if smoke; knowledge of nicotine patches so can deliver service well
**Skills**<br>*An ability or proficiency acquired through practice*	Skills; Skills development; competence; ability; interpersonal skills; practice; skill assessment	Communication skills
**Memory, attention and decision processes**<br>*The ability to retain information, focus selectively on aspects of the environment and choose between two or more alternatives*	Memory; attention; attention control; decision making; cognitive overload/tiredness	Remembering to deliver different parts of service
**Behavioural regulation**<br>*Anything aimed at managing or changing objectively observed or measured actions*	Self-monitoring; breaking habit; action planning	Are there systems which help monitor whether something has been done or not
**Environmental context and resources**<br>*Any circumstance of a person's situation or environment that discourages or encourages the development of skills and abilities, independence, social competence and adaptive behaviour*	Environmental stressors; resources/material resources; organisational culture/climate; salient events/critical incidents; person x environment interaction; barriers and facilitators	The lack of a consulting room impedes consultations
**Social influences**<br>*Those interpersonal processes that can cause individuals to change their thoughts, feelings or behaviours*	Social pressure; social norms; group conformity; social comparisons; groups norms; social support; power; intergroup conflict; alienation; group identity; modelling	Senior pharmacist not prioritising smoking cessation
**Professional/social role and identity**<br>*A coherent set of behaviours and displayed personal qualities of an individual in a social or work setting*	Professional identity; professional role; social identity; identity; professional boundaries; professional boundaries; professional confidence; group identity; leadership; organisational commitment	Identification that behaviour change is within a pharmacists remit
**Beliefs about capabilities**<br>*Acceptance of the truth, reality or validity about an ability, talent or facility that a person can put to constructive use*	Self-confidence; perceived competence, self-efficacy; perceived behavioural control; beliefs; self-esteem; empowerment; professional confidence	Confidence that can raise question of smoking in non-threatening manner
**Optimism**<br>*The confidence that things*	Optimism, pessimism; unrealistic optimism; identity	Belief that with support most people can give up smoking

will happen for the best or that desired goals will be attained		
**Beliefs about consequences**<br>*Acceptance of truth, reality, or validity about outcomes of a behaviour in a given situation*	Beliefs; outcome expectancies; characteristics of outcome expectancies; anticipated regret; consequents	If don't stop smoking will die from cancer
**Intentions**<br>*A conscious decision to perform a behaviour or a resolve to act in a certain way*	Stability of intentions; stages of change model; transtheoretical model and stages of change	Intention to give up smoking
**Goals**<br>*Mental representations of outcomes or end states that an individual wants to achieve*	Goals (distal/proximal); goal priority; goal/target setting; goals (autonomous/controlled) ; action planning; implementation intention	Wanting to achieve increased uptake by x amount
**Reinforcement**<br>*Increasing the probability of a response by arranging a dependent relationship, or contingency, between the response and a given stimulus*	Rewards (proximal/distal, valued/not valued, probable/improbable); incentives; punishment; consequents; reinforcement; contingencies; sanctions	Financial reward
**Emotion**<br>*A complex reaction pattern, involving experiential, behavioural and physiological elements by which the individual attempts to deal with a personally significant matter or event*	Fear, anxiety; affect; stress; depression ; positive/negative affect; burn-out	Discussing smoking raises distress due to family members recent smoking related death

Definitions of the COM-B model.

Capability

i) Physical Capability e.g. not having lost voice during smoking cessation consultation,

ii) Psychological Capability e.g. having knowledge of NRT products to talk about them

Opportunity

i) Physical Opportunity afforded by environment involving time, resources, locations, cues e.g. pharmacist having sufficient time to speak with clients

ii) Social Opportunity afforded by interpersonal influences, social cues, cultural norms influence way think about things e.g. pharmacist allows counter assistant time to become trained in stop smoking

Motivation

i) Reflective Motivation involving plans (intentions) and evaluations (beliefs about what is good and bad) e.g. intention to give up smoking because believe bad for health

ii) Automatic Motivation includes automatic processes which involve emotional reactions, desires (wants and needs), impulses, inhibitions, drive states and reflex responses.
